# Structural properties of Bi thin film grown on Si (111) by quasi-van der Waals epitaxy

**DOI:** 10.1038/s41598-022-06472-5

**Published:** 2022-02-17

**Authors:** Chieh Chou, Bo-Xun Wu, Hao-Hsiung Lin

**Affiliations:** 1 Graduate Institute of Electronics Engineering, National Taiwan University, Taipei, 10617 Taiwan; 2 Department of Electrical Engineering, National Taiwan University, Taipei, 10617 Taiwan

**Keywords:** Structural properties, Two-dimensional materials

## Abstract

Crystallinity of an 80-nm-thick bismuth thin film grown on Si (111) substrate by MBE was investigated. The highly (0003) textured Bi film contains two twinning domains with different bilayer stacking sequences. The basic lattice parameters c and *a* as well as b, the bilayer thickness, of the two domains were determined from a series of X-ray diffraction (XRD) measurements, and found that the differences are within 0.1% as compared with those of bulk Bi reported in literature, suggesting that the Bi film has been nearly fully relaxed. From the XRD φ-scans of asymmetric Bi (01–14), (10–15), (11–26) planes and Si (220) plane as well as selected area electron diffraction patterns and electron back scatter diffraction pole figures, we confirmed the well registration between the lattices of Si and Bi lattice, i.e. the ω angle difference between Bi[0003] and Si[111] and the φ angle difference between Bi[01–14] and Si[220] are 0.056° and 0.25°, respectively, and thus concluded that the growth is a quasi-van der Waals epitaxy.

## Introduction

Bismuth is an unusual semi-metal with a highly anisotropic Fermi surface and a very narrow inter-band overlap. Its intrinsic carrier concentration at room temperature is ~ 10^18^/cm^3^, just slightly higher than that of the narrow gap semiconductor InSb. Because of the small effective mass in certain orientations and the low intrinsic carrier density, using quantum-size effect to realize the semi-metal/semiconductor transition in Bi thin-film has drawn attentions for decades^1,2^. In addition, recent researches on bismuthene or ultrathin Bi layers observed peculiar surface properties such as metallic surface states formed by spin-orbital interaction^3,4^, quantum spin Hall effect^5,6^, and strain induced trivial-to-topological transition^5,7^, revealing that the potential applications of Bi and Bi-based materials to the magnetic devices. However, both the researches and applications of the versatile Bi properties depend mainly on the well-ordered structures, and the growth of nanoscale Bi thin films with high quality is of great importance.

**Figure 1:**
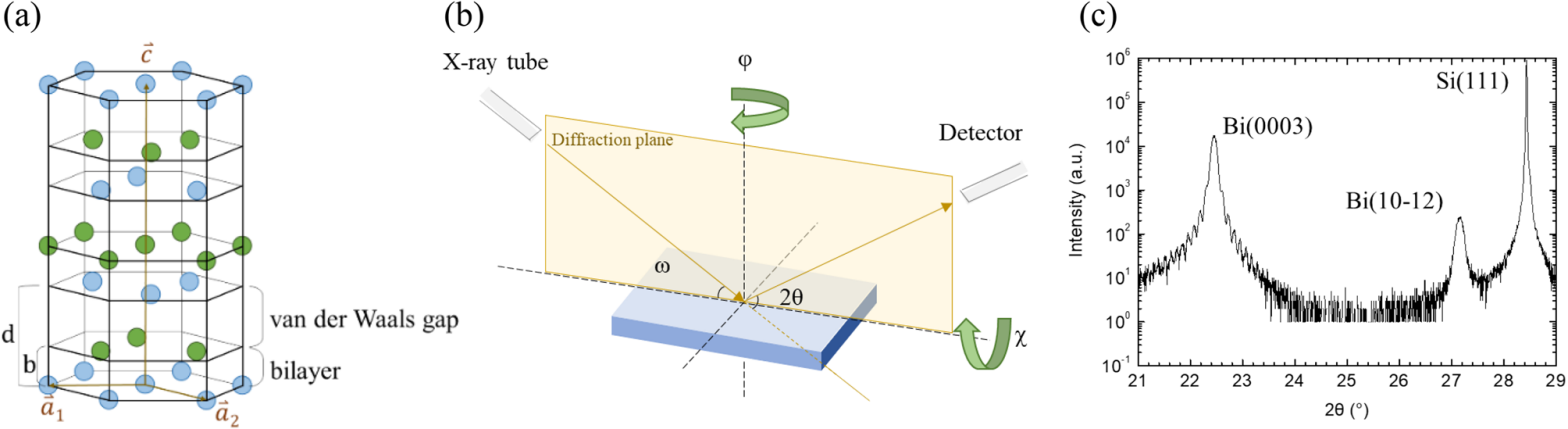
(**a**) Schematic diagram of rhombohedral Bi lattice drawn in a hexagonal lattice. The two basis atoms are represented by light blue and light green balls, respectively. Parameter b and d are the bilayer thickness and the distance between two adjacent bilayers, respectively. (**b**) Schematic diagram of the HRXRD measurement setup. (**c**) ω-2θ scan result of Bi thin film deposited on Si (111). The thickness of the film is around 80 nm calculated from the thickness fringe. *(a), (b) were created using Microsoft Office 2019 Pro Plus—URL: https://docs.microsoft.com/en-us/deployoffice/office2019/overview.

The lattice of bulk Bi is a layered structure. Each Bi atom covalently bonds three others to form a hexagonal bilayer network and the bilayers stack in ABC closed pack sequence along its trigonal direction (c-axis), shown in Fig. 1a. Atoms in the adjacent bilayers still have covalent charges to form a much weaker “semi-covalent” bonding^8^ or van der Waals bonding^9^. The coexistence of different bonding in the lattice may result in difficulties in the epitaxial growth. However, the weaker bilayer bonding also allows the growth through the van der Waals bonding interfaces to mitigate the effect of biaxial strain resulting from the huge lattice mismatch, on the crystallinity.

Various substrates including Si, BaF_2_ and glass have been used for the growth of Bi thin film^2,6–8,10–14^. Among them, Si substrate is of great importance because it is the platform of integrated circuits. The feasibility of utilizing semimetal to semiconductor transition through quantum confinement effect to realize Bi semiconductor devices or all Bi semimetal/semiconductor junction device^15,16^ as well as Bi semimetal contact on MoS_2_ 2D FET with ultralow resistance^17^ have been demonstrated very recently. For the growth of Bi thin film on the (111) close-packed plane of Si, a previous work utilized in-situ low energy electron diffraction technique to observe a structure transition from disordered pseudo-cubic Bi (110) grains into twinned hexagonal Bi (111) grains^10^ when the coverage of Bi is 7 monolayers. Other studies demonstrated the growth of two sets of twinned Bi (111) grains on (100) Si substrate^11,12^. Though the plane is not a close-packed plane, the well-ordered domains orient with respect to the Si substrate. These works reveal the successful quasi-van der Waals epitaxial growth of Bi (111) on both (111) Si and (100) Si substrates. However, very few reports on the in-plane structure or the granular properties of Bi thin films^14^. In this work, we present a detailed study on the structural properties of a Bi thin film grown on Si (111) substrates by molecular beam epitaxy (MBE). Bi lattice parameters including *a*, c, and b, the bilayer thickness, were determined by high-resolution X-ray diffraction (HRXRD) on different planes. The registry between Bi and Si lattice was checked by HRXRD φ-scan and selected area electron diffraction (SAED) and electron back scatter diffraction (EBSD). Twinning and granular properties in Bi thin film were also investigated using HRXRD φ-scan, SAED and EBSD.

## Results and discussion

**Figure 2:**
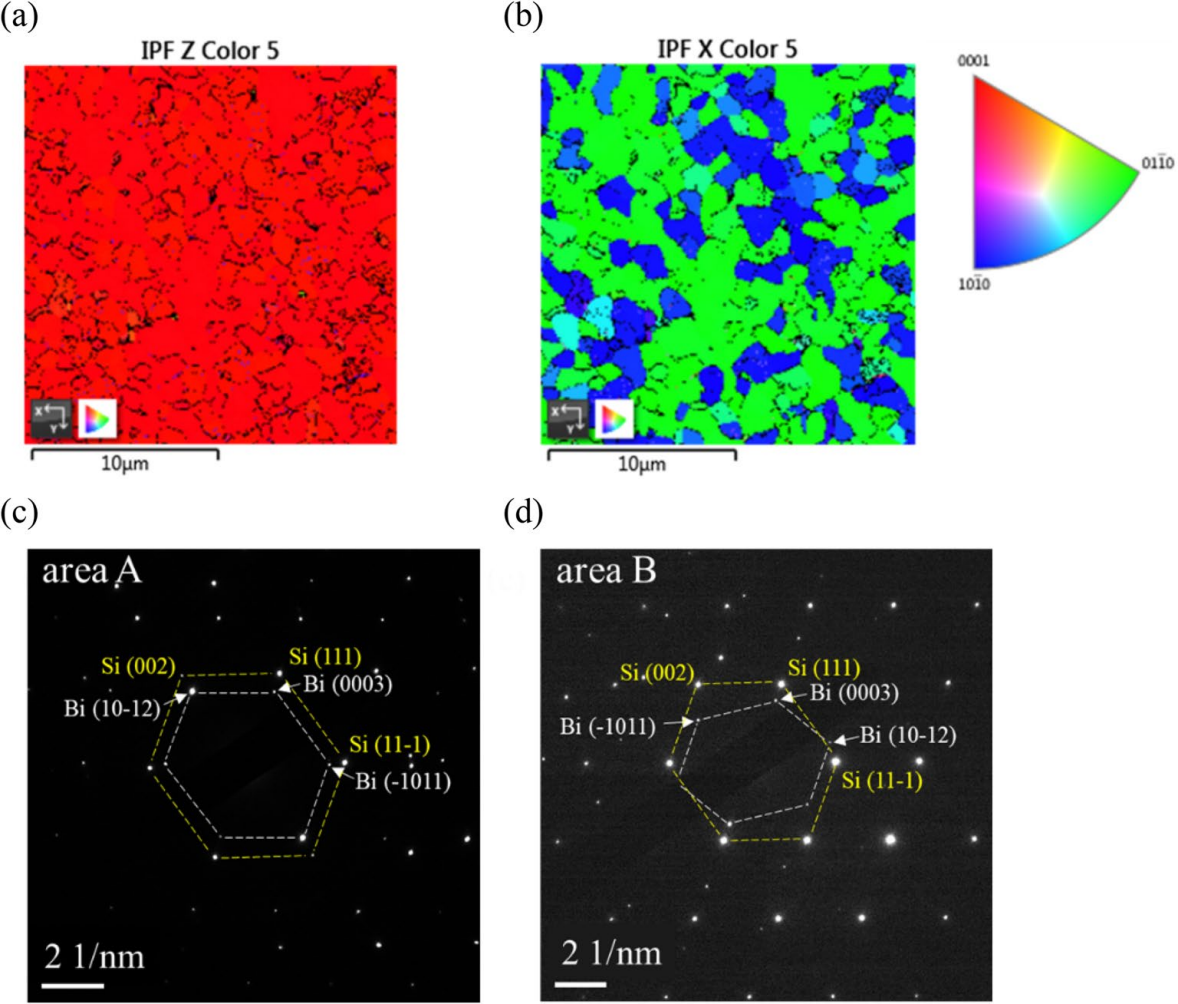
(**a**) IPF Z mapping and (**b**) IPF X mapping of Bi film obtained from EBSD measurement. The crystal orientation along the specified direction is indicated by the colored sector. (**c**) TED images performed at area A and (**d**) area B. The yellow hexagons are connected by Si diffraction spots while the white hexagons are connected by Bi spots.

Figure 1b shows the setup of HRXRD with the angles which will be referred hereafter. Figure 1c shows the result of ω-2θ HRXRD scan of a Bi thin film with a thickness of ~ 80 nm, deposited on Si (111) substrate by molecular beam epitaxy (MBE). In the figure, the strong peak at 22.45° is from the diffraction of Bi (0003) planes. The FWHM Δ(2θ) of the peak is 0.11°, close to the value calculated from Scherrer equation for 80 nm thickness. Besides, the clear thickness fringes around the peak indicate that the film has a rather smooth surface. These findings reveal that the growth is mainly along the c-axis with a well-ordered sequence, despite the ~ 18% lattice mismatch between Bi and Si. The peak at 27.16° is the diffraction of Bi (10–12) planes. The existence of this peak reveals the granular in-plane structure of the Bi film. To understand the in-plane structure, we performed electron backscatter diffraction (EBSD) measurement and the inverse pole figure (IPF) Z mapping and IPFX mapping are shown in Fig. 2a and b, respectively. The orientation can be identified by the colored circular sector with the coordinate system for hexagonal lattice. As shown in Fig. 2a, the whole region is almost red except some black spots, indicating that the Bi film is [0003] textured. The black spots are located at the grain boundary where the defects make the orientation unresolvable. The (10-12) phase (purple or yellow color), however, is not visible in Fig. 2a. In Fig. 1c, the intensity ratio of (10-12) to (0003) is 1/60. Considering that the structure factor of (10-12) is about 10 times larger than that of (0003), the probability ratio of appearing (10-12) grain to (0003) grain is ~ 1/1600. Since the IPF mappings cover only several hundred grains as shown in Fig. 2b, the grain of (10–12) could omit this measurement area due to its low probability. The IPFX mapping, shown in Fig. 2b, clearly depicts grains with sizes in several microns. Almost all the grains are either in grass green or in deep blue except several scattered grains in light blue. The number of grass green is much more than that of deep blue, indicating that [01-10] represented by grass green is the preferential direction. The second largest color is along [10-10] which is 60º with respect to [01-10]. To further elucidate the relation between the lattices of Bi twinning phases and the Si substrate, the TED images took from two different twinning phases are shown in Fig. 2c and d, respectively. In Fig. 2c, we can see that the two hexagons are similar and with the corresponding sides in parallel. However, in Fig. 2d, though the two hexagons are similar too, the smaller one is the refection of the smaller hexagon of Fig. 2c with respect to the (0003) axis. These results indicate that the two twinning Bi grains are epitaxially grown on Si substrate, and the Bi epitaxial films are nearly fully relaxed. From the TED image of Fig. 2c, it is interesting that the forbidden (002) and (00-2) spots of Si are much fainter than other Si spots. While the (10-12) and (-101-2) Bi spots, near to the forbidden Si spots are much brighter than other Bi spots, which is due to their strong structure factor.

**Figure 3:**
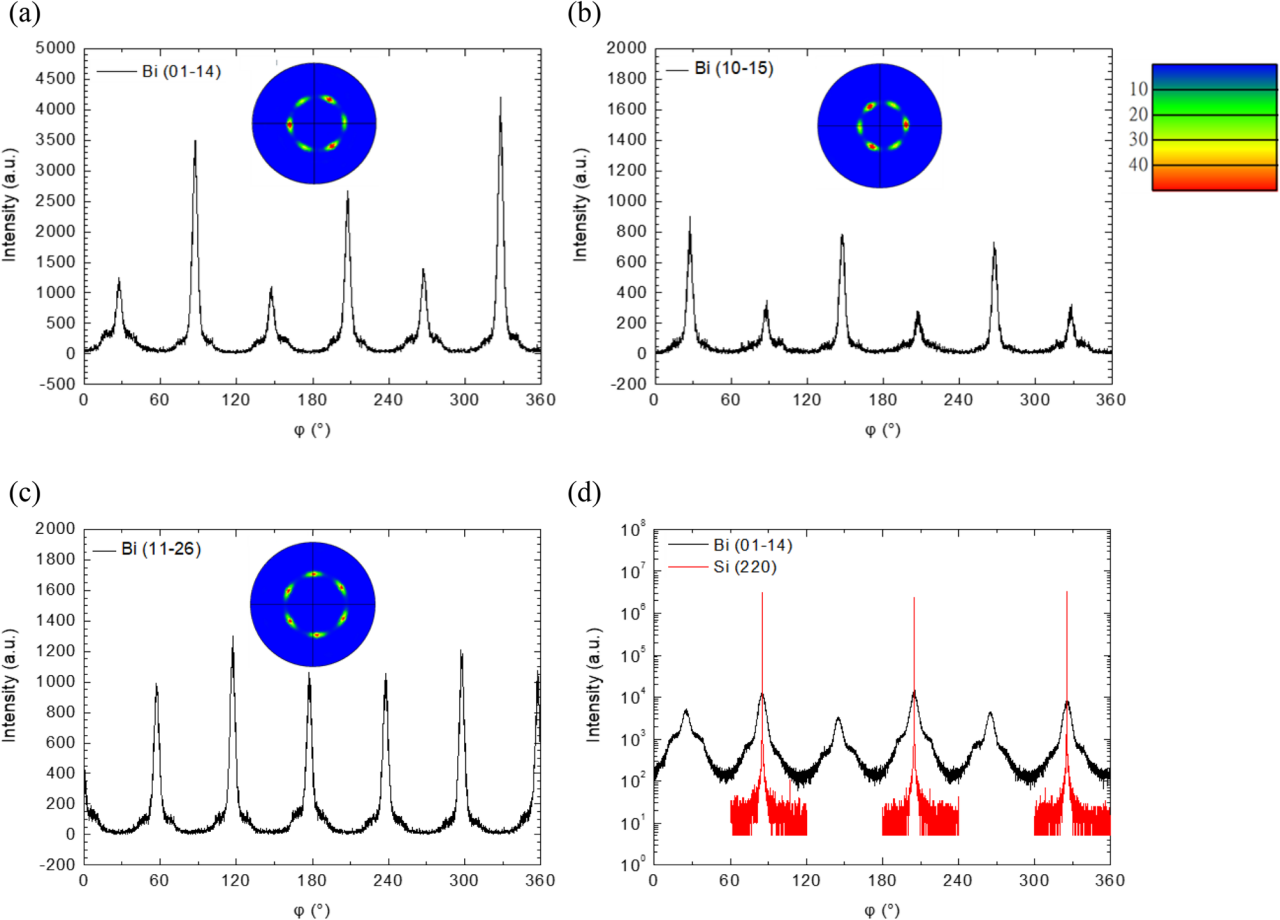
HRXRD φ scans of asymmetric (**a**) Bi (01-14), (**b**) Bi (10-15), (**c**) Bi (11-26) planes. The EBSD pole figures of the three Bi planes are shown as the inset in their panels with the intensity color bar shown in the right top of (**b**). (**d**) HRXRD φ scans of Si (220) and Bi (01–14) planes in logarithmic scale.

To further understand the relationship between the Si substrate and the preferential growth of the Bi film, we performed XRD measurements for the tilting planes (01-14), (10–15), and (11-26) of the Bi twinning phases as well as the (220) planes of the Si substrate. Note that a trigonal lattice becomes an FCC lattice when its lattice constants have the relation: c = $\sqrt 6$ *a*. In this situation, {01-14} of the hexagonal trigonal system is equivalent to the {220} of the cubic FCC system, and the c/*a* of Bi is larger than $\sqrt 6$ by only ~ 6.5%. In here, we compare the φ-scans of Bi (01-14) and Si (220) to understand which twinning phase follows the stacking sequence of Si. The φ-scan of the four tilting planes are shown in Fig. 3a–d, respectively. In the measurement, the [11-2] of the Si substrate parallels to the diffraction plane when φ = 0°. For the measurement of (01-14), (10-15), and (220) planes, the sample was rotated to φ = 90° to let the projection of the plane normal on the sample surface perpendicular to the diffraction plane. Then, the plane for measurement was tilted with a χ angle to let its normal on the diffraction plane to perform ω-2θ scan. The schematic diagram for the aforementioned procedures can be found in Supplementary Fig. S1 online. After a fine-tuning procedure, we obtained the best Bragg’s angle, at which φ-scan was performed. The same procedures were used for (11-26) plane except that the initial φ angle was set at 60°. As shown in Fig. 3a and b, there are 3 stronger peaks interleaved by 3 weaker peaks. The results of (01-14) and (10-15) planes, obviously, in good agreement with the EBSD polar figures shown as insets in the same figures. Note that the two planes, (01-14) and (10-15) planes, have threefold rotation symmetry but with 60° phase shift to each other. The stronger 3 peaks belong to the preferential twinning phase that well resembles the bottom Si substrate, and the weaker 3 peaks belong to the other twinning phase. Plane (11-26), however, is with sixfold rotation symmetry. The 6 peaks are the superposition of the two twinning phases and with nearly the same peak intensities as shown in Fig. 3c, which is in good agreement with the EBSD polar figure shown as an inset in the same figure. In Fig. 3d, the φ-scans of Bi (01-14) and Si (220) are depicted together for comparison. As can be seen, the Si peaks well meet the three stronger Bi peaks with the φ angle difference within 0.25°, indicating that the preferential Bi (0003) phase is indeed with the same stacking sequence with the Si substrate. Although the Bi (0003) plane contains a van der Waals gap, the bottom Si substrate still affects the stacking sequence of the epitaxial growth. The mechanism is not understood yet. However, from the titling direction of the Bi film which will be discussed later, the terrace steps could play a role for this effect. From Fig. 3d, due to the logarithm scale in intensity, one can see that the Bi peaks are all with a low intensity skirt, implying the existence of a minor group of grains with larger deviation in in-plane lattice, which could result from the large mismatch between Bi and Si in lattice parameter *a*.

**Figure 4:**
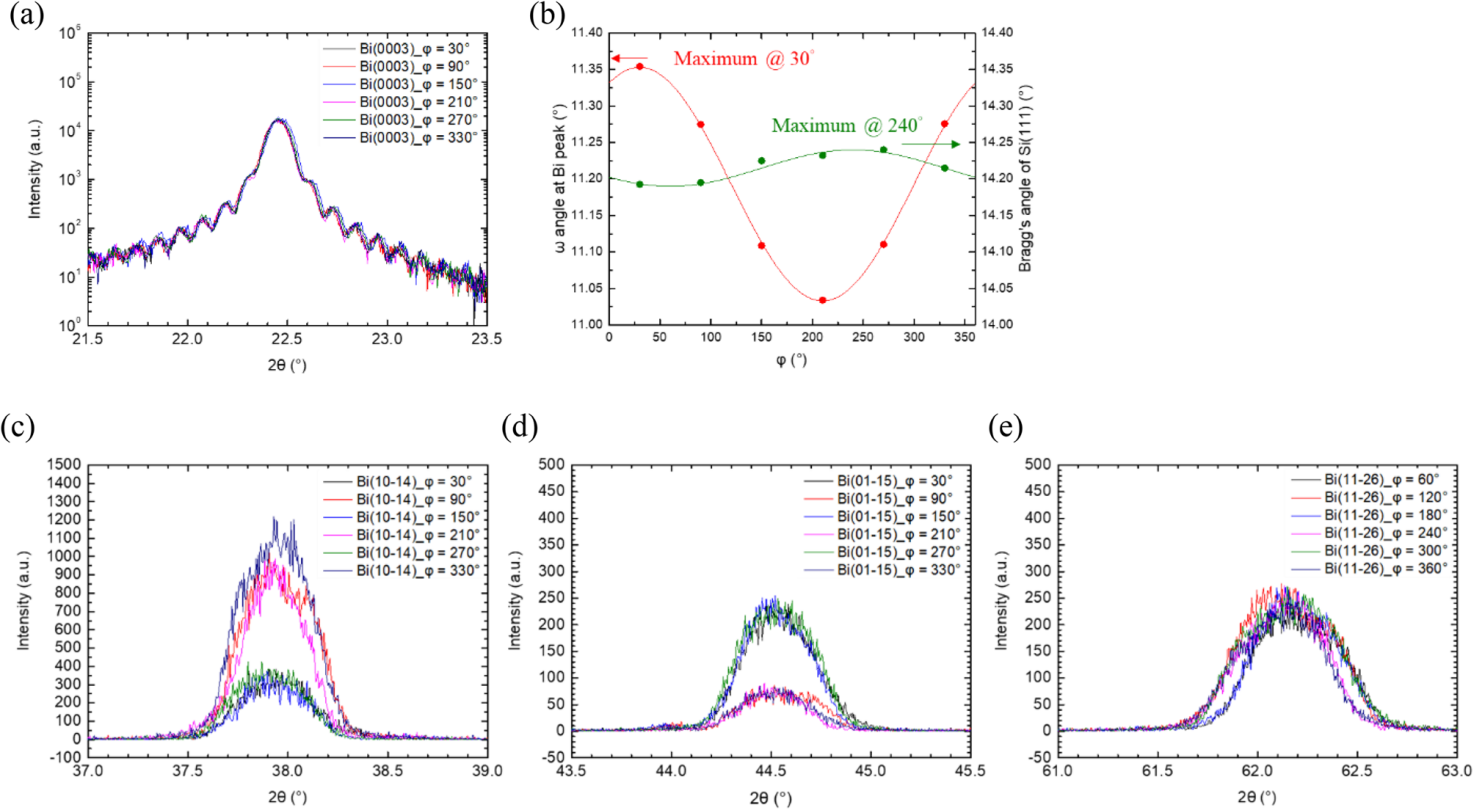
(**a**) HRXRD ω-2θ scans of Bi (0003) at six different φ angles. (**b**) The oscillation behavior of Bi (0003) ω-angles and Si (111) Bragg’s angle for different φ angles. ω-2θ scans of asymmetric (**c**) Bi (01-14), (**d**) Bi (10-15), (**e**) Bi (11–26) planes are also performed at six different φ angles.

Figure 4a shows the ω-2θ scans of Bi (0003) at six different φ angles as indicated in the figure. In each measurement, Ω and χ angles were fine-tuned to obtain the best scan. The curves almost coincide and the FWHM is ~ 0.11°. Figure 4b shows the ω angle at the peak and the Bragg’s angle of Si (111) as functions of φ angle. From the oscillations of the angles, we can find the relationships between the normal of the substrate surface, Bi (0003), and Si (111). As can be seen, the oscillation of ω angle is with an amplitude of 0.16° and reaches its maximum when φ = 30°, indicating that the normal of Bi (0003) tilts toward the [10–1] direction of Si substrate by 0.16°. On the other hand, the oscillation of the Bragg’s angle of Si (111) is with an amplitude of 0.056° and reaches the maximum at 240°, indicating that the normal of Bi (0003) tilts toward the [2-1-1] direction of Si substrate by 0.056°. The relation between the XRD φ angle and the in-plane Si (111) substrate direction can be found in Supplementary Fig. S2 online. Note that the step edges are perpendicular to the [2-1-1] direction on Si (111)^18^. Although the Si substrate is slightly mis-orientated, the tilting direction of the Bi film suggests that it is still affected by the steps on the Si substrate.

The ω-2θ scans of Bi (01-14), Bi (10-15) and Bi (11-26) are shown in Fig. 4c–e. The scans were performed at six different φ angles, indicated in the figures. In contrast to Bi (0003), the Bragg’s angles of the three planes are in much larger variation for different φ angles and the FWHM’s are also much broader. For some cases, the Bragg’s reflection clearly can be decomposed into two peaks resulting from two groups of grains, which is consistent with the low intensity skirt observed from the φ-scan of plane (01-14) shown in Fig. 3d. Therefore, we conclude that the vertical structure of Bi film is in much better order than its in-plane structure. The latter clearly contains different grains and could suffer from defects and strains resulting from huge lattice mismatch between Bi and Si substrate as well as the defects at grain boundaries.

**Figure 5:**
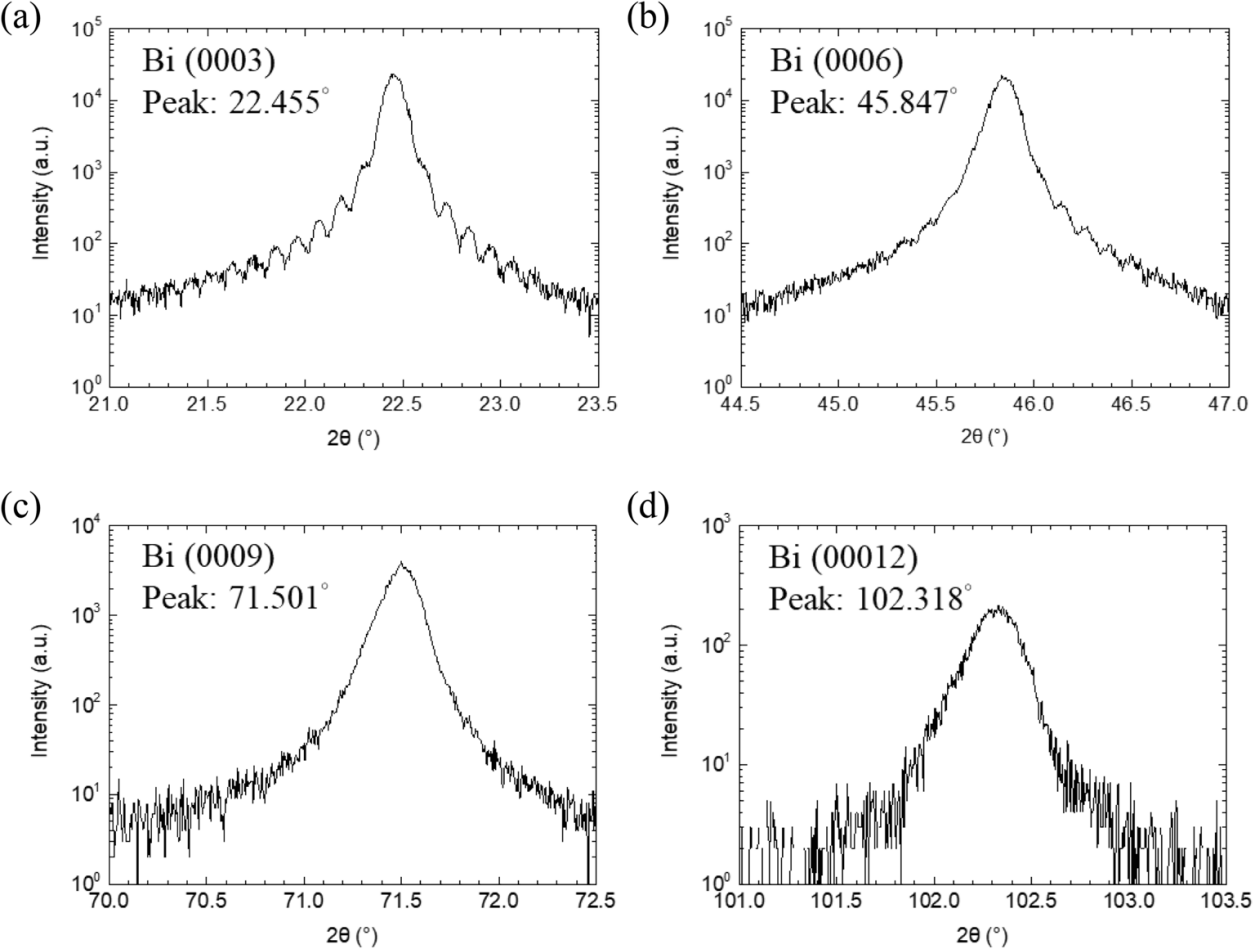
HRXRD ω-2θ scan results of (**a**) Bi(0003), (**b**) Bi(0006), (**c**) Bi(0009), (**d**) Bi(00012) planes.

The determination of the lattice parameter *a* and c allows us to understand more detailed structural properties. Since the vertical structure of the Bi film is in much better order, we performed XRD measurement on (0003), (0006), (0009), and (00,012) to determine c parameter first. The results are shown in Fig. 5. From their Bragg’s angle, we can find the period of the plane and determine the c parameters. The average and standard deviation of c parameters are 11.870 Å and 0.0015 Å, respectively. The values are listed in Table 1. Then, we used the Bragg’s angles obtained from Fig. 4c–e to find the plane period and utilized the following Eq. (1) to calculate *a* parameters. The equation expresses the relation between plane period d_(pqrs)_ and lattice parameters *a* and c for a plane with hexagonal Miller indices (h k l m),

1 $$\frac{1}{{{\text{d}}_{{\left( {hklm} \right)}}^{2} }} = \frac{4}{3}\frac{{{\text{h}}^{2} + {\text{hk}} + {\text{k}}^{2} }}{{a^{2} }} + \frac{{{\text{m}}^{2} }}{{{\text{c}}^{2} }}$$

**Table 1:** Summary of room temperature lattice parameters of our Bi nanofilm. Bulk single crystal results^20^ are listed for comparison.

Lattice parameters	*a* (Å)	c (Å)	c/*a*	b/d	B (Å^2^)
C. S. Barrett	4.546 $\pm$ 0.0005	11.862 $\pm$ 0.001	2.609	0.40408 $\pm$ 0.00047	1.144
This work	4.545 $\pm$ 0.001	11.870 $\pm$ 0.003	2.612	0.40388	1.868

The lattice parameter c was set as the average value listed in Table 1 and the parameter *a* can thus be uniquely determined for each d_(hklm)_. In total, we have 18 measurements for 3 different titling planes at 6 different φ angles. The average and standard deviation for *a* are 4.545 Å and 0.0096 Å, respectively. The values are also listed in Table 1. For comparison, the lattice parameters determined from Barret^19^ are also listed in Table 1. Barret’s results were determined from zone-refining Bi single crystal and are considered free from defects and strains. Our parameter *a* is very close to Barret’s value. However, the difference is much smaller than our standard deviation and further discussion in *a* becomes inappropriate. But the difference between two c parameters is larger than our standard deviation. Therefore, we believe that the epitaxial Bi thin film is nearly fully relaxed but might with a very small in-plane compressive stain in the range of 10^–4^. Kammler and Horn-von Howgen estimated 2% compressive strain in the 7 ML bismuth film deposited on Si (111) from their LEED observation^10^. For the Bi film grown on Si (100), the estimated strain from LEED was also 2.4% for 3 ~ 4-nm-thick Bi film^12^. In contrast, our 80-nm-thick film is about 200 ML and has been nearly fully relaxed. Both results indicate the effect of van der Waals gap on the heterepitaxy.

In addition to lattice parameters c and *a*, the bilayer thickness, b, is also an important parameter which cannot be directly determined from XRD measurements. Notice that the top atoms and bottoms atoms in a bilayer belong to different basis atoms as shown in Fig. 1a. Therefore, the bilayer thickness affects the structure factor of the lattice and thus the integrated XRD intensity. In this work, we derive the thickness by comparing the integrated XRD intensities of different planes. However, the integrated intensity is also a function of Bragg’s angle, θ, and Debye–Waller factor which contains average mean square of atomic displacement. In here, we list a simplified formula (2) ^20^, considering only the terms relevant to bilayer thickness, b, Bragg’s angle, θ, Debye–Waller factor, e^−2 M^, and the Miller indices of the (h k l m) plane, for the integrated XRD intensity, I, as follows,

2 $${\text{I}} = {\text{Ae}}^{{ - 2{\text{M}}}} {\text{f}}_{{\text{j}}}^{2} {\text{LA}}_{c} \left\{ {1 + {\text{cos}}\left[ {\frac{{2{\uppi }}}{3}\left( {1 + \frac{{\text{b}}}{{\text{d}}}} \right){\text{s}}} \right]} \right\}$$

where d is the distance between two adjacent bilayers which is c/3, A is a constant independent of Bragg’s angle and orientation, f_j_ is the atomic scattering factor, L is the Lorentz factor, and A_c_ is the absorption correction factor. The details of the parameters are described as follows.

For the Debye–Waller factor, e^−2 M^, the factor M is proportional to the average mean square displacement of the atoms to their lattice points along the normal of plane (h k l m). The atomic displacement is due to thermal vibration and lattice imperfections. In here, because of the aforementioned severe disorder in tilting planes, which could hinder the obtaining of accurate intensity, we consider only (0 0 0 m) planes. We thus use the following equation to express the M factor^21^,

$${\text{M}} = 8\pi ^{2} \left( {\frac{{\sin ~\theta }}{\lambda }} \right)^{2} \left[ {\left\langle {\overline{{{\text{r}}_{ \bot }^{2} }} } \right\rangle \left( {\frac{{{\text{s}}^{2} }}{{{\text{c}}^{2} }}} \right)} \right] = \left( {\frac{{\sin ~\theta }}{\lambda }} \right)^{2} \left[ {{\text{B}}\left( {\frac{{{\text{s}}^{2} }}{{{\text{c}}^{2} }}} \right)} \right]$$

where $\left\langle {\overline{{{\text{r}}_{ \bot }^{2} }} } \right\rangle$ is the vertical average mean square atomic displacement and B = 8π^2^ $\left\langle {\overline{{{\text{r}}_{ \bot }^{2} }} } \right\rangle$. Atomic scattering factor of Bi atom, f_j_, is a function of Bragg’s angle and wavelength. The value used in this work was calculated using the polynomial formula from Ref.^21^. The Lorentz factor, L, includes the polarization factor and the angular velocity. Our XRD has a Ge (220) first crystal and L is given by,

$${\text{L }} = { }\frac{{1 + {\text{cos}}^{2} 2{\uptheta }_{{\text{M}}} {\text{cos}}^{2} 2{\uptheta }}}{{{\text{sin }}2{\uptheta }}}$$

where θ_M_ is the Bragg’s angle for Ge (220) and cos 2θ_M_ = 0.7033. The Cu-Kα absorption coefficient of Bi, μ, is 2391 /cm and the film thickness is only 80 nm, the effect of absorption must be considered, the correction factor A_c_ is given by

$${\text{A}}_{{\text{c}}} = 1 - {\text{exp}}\left( {\frac{{ - 2{\mu t}}}{{{\text{sin }}\uptheta }}} \right)$$

Finally, we may obtain a function F(b/d) by dividing Eq. (2) by the measured X-ray intensity, I_exp_. The function is expressed as,

3 $${\text{F}}\left( {\frac{{\text{b}}}{{\text{d}}}} \right) = \frac{{{\text{I}}/{\text{A}}}}{{{\text{I}}_{{{\text{exp}}}} }} = \frac{{{\text{e}}^{{ - 2{\text{M}}}} {\text{f}}_{{\text{j}}}^{2} {\text{LA}}_{{\text{c }}} \left\{ {1 + {\text{cos}}\left[ {\frac{{2{\uppi }}}{3}\left( {1 + \frac{{\text{b}}}{{\text{d}}}} \right){\text{s}}} \right]} \right\}}}{{{\text{I}}_{{{\text{exp}}}} }}$$

**Figure 6:**
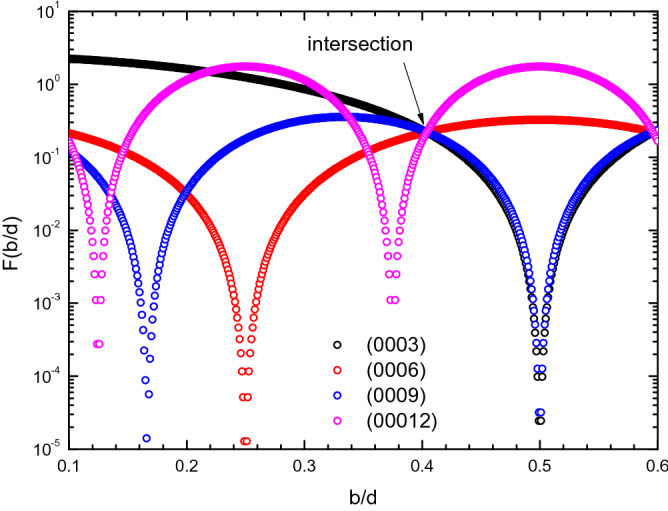
F(b/d) versus b/d plots for Bi (0 0 0 s) planes. The curves intersect at one point when b/d is 0.4039.

Intuitively, the F value of different planes should be the same if we select correct b/d value and Debye–Waller factor. In addition to b/d and Debye–Waller factor, the final F value is also unknown. We thus need at least three planes to find the three unknown variables. We selected the integrated XRD intensities of (0006), (0009) and (00,012) planes to resolve the b/d, B, and F(b/d). The F(b/d) versus b/d curves for (0003), (0006), (0009), and (00,012) are depicted in Fig. 6. As can be seen the curves intersect at a point where b/d = 0.4039 and F(b/d) = 0.2203. The solved B for the Debye–Waller’s factor is 1.868. The results are listed in Table 1. In the solving, (0003) plane was not chosen because of the extinction effect resulting from its small Bragg’s angle, suggested in Ref. 19. However, as shown in Fig. 6, the curve of (0003) is also very close to the point of intersection. Its F(b/d) value is slightly lower than that of the point of intersection by 0.9%. Table 1 also lists the b/d and B values reported by Barret. Our b/d is very close to Barret’s value. For the B values, ours is larger than Barret’s value by 63%^22^. Barret’s results were obtained from a Bi single crystal produced by zone-refining method and the B value is believed mainly from thermal vibration. In contrast, our B value is from an epitaxial Bi thin-film, must suffer from defects and strains resulting from the huge lattice mismatch as well as the granular in-plane structure. However, the b/d value is close to the value of single crystal and the B value is low despite the aforementioned in-plane disorder. We believed that the van der Waals gap must play an important role.

## Conclusion

In conclusion, the lattice structure of an 80-nm-thick Bi thin film grown on (111) Si substrate by MBE has been investigated. Most of the grains in the film were grown along [0003] direction but with two different stacking sequences, i. e., in two twining phases. From EBSD, XRD φ-scan, and SAED measurements, we found that the preferential twining phase well register the lattice of the bottom Si substrate. The lattice parameters, c, *a*, and b as well as the Debye–Waller factor of the (0003) grains have been determined. The parameters are close to those of bulk Bi single crystal, implying that the grains are nearly fully relaxed despite the huge ~ 18% lattice mismatch. From these findings, we believe that the growth is a quasi-van der Waals epitaxy.

## Methods

### MBE of nanoscale Bi

Bi thin film was deposited on Si(111) substrate by MBE. Before the growth, Si wafers were degreased in acetone, methanol and isopropyl alcohol each for 2 min and then soaked into 2% HF solution for 1 min to remove the native oxide. The wafers were then loaded into SVTA MBE system and were baked at 300 °C under UHV environment for 1 h in the buffer chamber and desorbed at 850 °C for 5 min in the growth chamber. The temperature of substrate was a critical parameter during the growth, which was fixed at 130 °C and the growth time was 30 min. Typical RHEED patterns before and after bismuth deposition can be found as supplementary Fig. S3.

### HRXRD spectra

HRXRD spectra of Bi film were carried out on Bruker New D8 Discover with an X-ray wavelength of 1.5406 Å (Cu K-α) and an integration time ranging from 0.1 s to 1 s.

### SAED pattern

SAED pattern of Bi grains were carried out on JEOL JEM-2010F TEM with an accelerating voltage of 200 kV.

### EBSD maps

EBSD maps of Bi film were performed on JEOL JSM-7800F PRIME with EBSD NordlysMax3 detector. The accelerating voltage is 20 kV.

## Supplementary Information

Supplementary Figures.
