# Supplementary material for: Structural properties of Bi thin film grown on Si (111) by quasi-van der Waals epitaxy
Source: Sci Rep. 2022 Feb 17;12:2764. doi: 10.1038/s41598-022-06472-5 (PMC8854617; doi:10.1038/s41598-022-06472-5)
Supplement: Supplementary file 1 — Supplementary Figures. [file 41598_2022_6472_MOESM1_ESM.pdf]

Supplementary Material

Structural properties of Bi thin film grown on Si (111) by quasi-van der Waals epitaxy

Chieh Chou[1], Bo-Xun Wu[1], and Hao-Hsiung Lin[1,2,*]

[1] *Graduate Institute of Electronics Engineering, National Taiwan University, Taipei, 10617, Taiwan*

[2] *Department of Electrical Engineering, National Taiwan University, Taipei, 10617, Taiwan*

*Corresponding author. Email address: *hhlin@ntu.edu.tw

1. XRD configurations for measuring tilting Si (220) plane

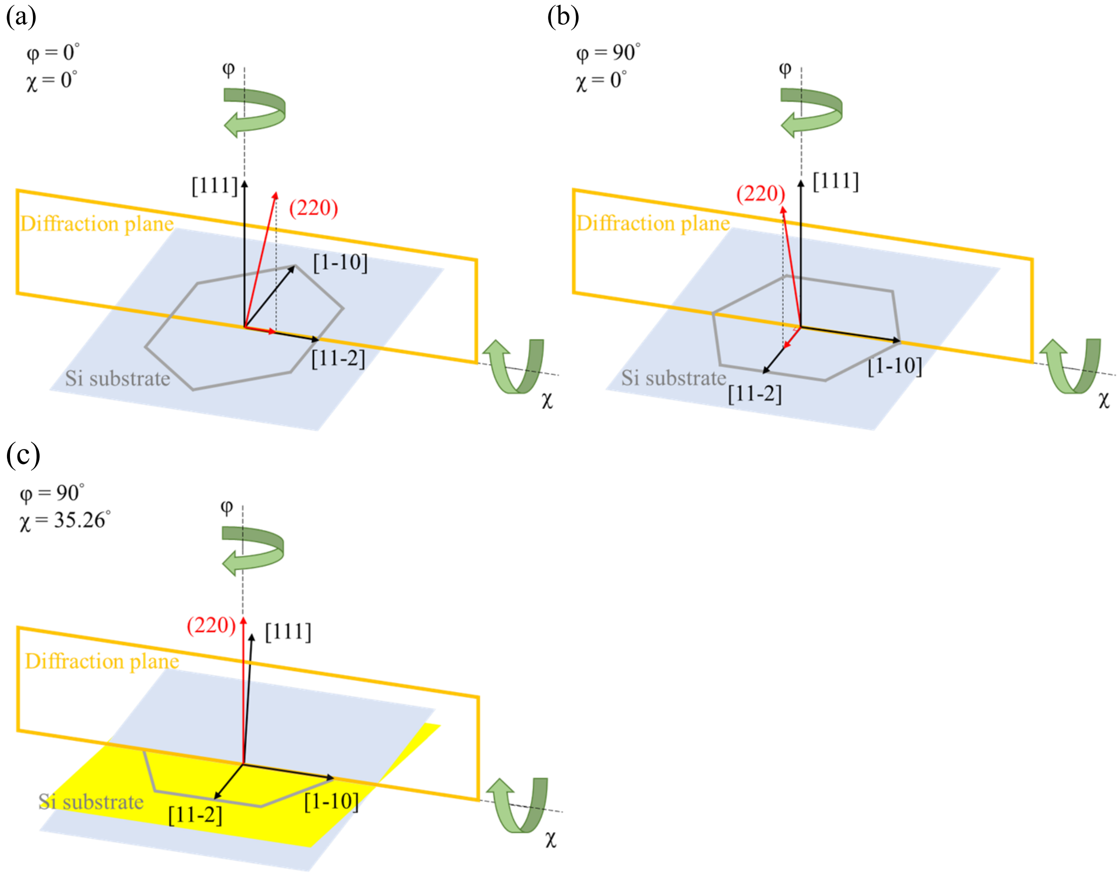

Figure S1. (a) Schematic diagram of the HRXRD configuration when φ angle is at 0˚. The [11-2] of the Si substrate parallels to the diffraction plane. (b) For the measurement of Si (220) planes, the sample was rotated to φ = 90°. The projection of (220) plane normal on the sample surface is perpendicular to the diffraction plane. (c) (220) plane was tilted with a χ angle to let its normal on the diffraction plane to perform ω-2θ scan. Here χ angle is 35.26˚, referred to the angle between Si (111) and (220) plane normal. XRD measurements for Bi (01-14), (10-15) and (11-26) plane follow same procedures. *All the figures were created using Microsoft Office 2019 Pro Plus - URL: https://docs.microsoft.com/en-us/deployoffice/office2019/overview.

2. Relation between XRD φ-angle and in-plane Si (111) substrate direction

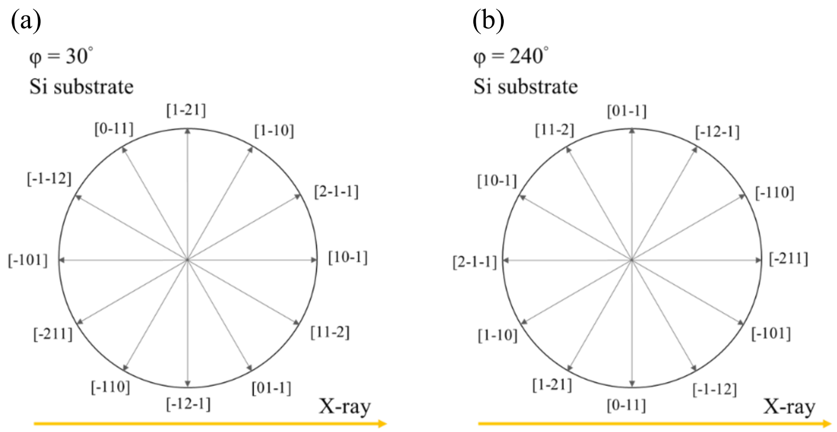

Figure S2. In-plane orientation maps of Si (111) substrate when φ angle is at (a) 30° and (b) 240°. Incident direction of the X-ray beam is indicated by yellow arrow. The orientation maps were determined by the primary orientation flat on the wafer and confirmed by XRD Si (220) scan. *All the figures were created using Microsoft Office 2019 Pro Plus - URL: https://docs.microsoft.com/en-us/deployoffice/office2019/overview

3. Typical RHEED patterns before and after bismuth deposition

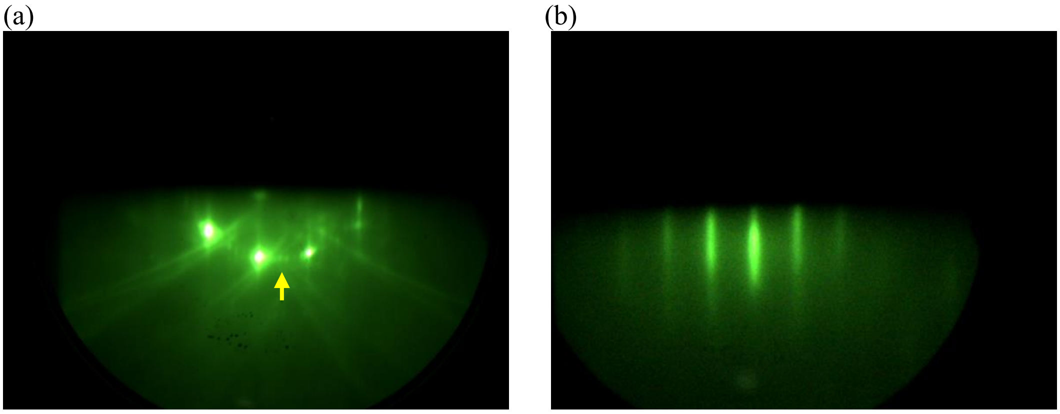

Figure S3. (a) Typical RHEED pattern of Si(111) after oxide desorption at 900°C and cooled down to 300°C. The 7×7 Si(111) surface reconstruction is indicated by the yellow arrow. (b) RHEED pattern after depositing ~30ML Bi on Si(111) substrates. The incident electron beam was nearly parallel to [1-10] azimuth of the Si(111) surface and with an energy of 6.5 keV. The ratio of the bulk line spacing in (a) to in (b) is ~1.17, roughly corresponding to the period ratio of Bi(11-20) to Si(2-20).
